# Sustainable Feeds for Animal Nutrition in Tropical Areas

**DOI:** 10.3390/ani13081379

**Published:** 2023-04-18

**Authors:** Bossima Ivan Koura, Maria Shipandeni, Monica Isabella Cutrignelli

**Affiliations:** 1 Ecole de Gestion et d’Exploitation de Système d’Elevage, Université Nationale d’Agriculture, Ketou P.O. Box 43, Benin; 2 Department of Animal Science, University of Namibia, Windhoek 13301, Namibia; tautiko@gmail.com; 3 Department of Veterinary Medicine and Animal Production, University of Napoli Federico II, Napoli, F. Delpino 1, 80137 Napoli, Italy

## 1. Introduction

Developing efficient feeds and sustainable feeding systems is required to meet the increasing demand for livestock and livestock products [1]. Therefore, one of the main objectives in animal nutrition is to explore alternative feeds and investigate their impact on animal performance and the quality of their products. In tropical areas, there are many opportunities to explore, including the use of herbs and legumes as feed additives and the improvement of grazing management on native heterogeneous vegetation.

We summarize the studies published in this Special Issue on recent advances in animal nutrition in tropical areas. These studies addressed the nutritional value assessment of climate-resilient forage and tree seeds, with a review of interventions to improve feed efficiency; alternative feeds and feed additives such as legumes seeds, leaves and processing by-products on fermentation characteristics, and methane production; animal performances and health; and meat quality.

## 2. Sustainable Feeds Investigated in This Issue

### 2.1. Ruminant

In tropical areas, rangelands with native heterogeneous vegetation, including grasses, legumes, trees, and shrubs, are the primary forage source for ruminants. Climate change increased the frequency of drought, resulting in fodder shortages during the dry season. Koura et al. [2] identified and assessed the nutritional value of thirteen drought-tolerant plants in the coastal grasslands of Benin in West Africa. Most of these forage plants, particularly Poaceae, were poor in nutritional value; however, the cultivation of some promising drought-tolerant plants such as *Dactyloctenium aegyptium*, *Zornia latifolia*, and *Chamaecrista rotundifolia*, which showed the best nutritional characteristics, could be used to sustain ruminant production along these coastal areas. This study permits authors to assess, for the first time, the nutritional value of *Andropogon virginicus*, *Cenchrus biflorus*, and *Z. latifolia*. However, further studies are required to investigate these species’ morphological traits and quality in cultivation conditions.

The potential of the tropical herbaceous plant, especially *Mitragyna speciosa* (Korth) Havil, was tested as a feed additive to assess the effect on ruminant performances and meat quality. Plant leaf powder has been found to be rich in phytonutrient elements that improved the hot carcass weight, longissimus muscle area, oleic acid content, and protein content of meat in growing goats [3]. Moreover, no detrimental impacts were found on the body weight, average daily gain, feed conversion ratio, and carcass composition of goat meat. The hot carcass weight, longissimus muscle area, oleic acid (C18:1n9), monounsaturated fatty acid (MUFA), and protein content increased, suggesting the Mitragyna leaf as a viable alternative feed additive in the future.

Processing by-products from another herbaceous plant, indigo (*Indigofera tinctoria* L.) waste from the processing of natural indigo dye, has been found to be a low-cost protein source for ruminants. Indigo waste allowed similar performances as concentrate diets [4]. Indeed, feed intake, digestibility, rumen fermentation, or growth performance in growing beef cattle were maintained without affecting hematology or immune function. However, low levels (10%) of this by-product should be used, and more research should investigate its effects on carcass characteristics and the meat quality of beef cattle.

The effect of twenty-two crude ethanolic plant extracts on the in vitro rumen fermentation of *Themeda triandra* hay was investigated. The microbial protein yield improved with all extracts, while some plants also increased the digestibility and utilization of poor forages [5]. Therefore, the expected positive impact on animal performances and methane mitigation on ruminants fed with poor forages need to be verified via in vivo studies.

Recent studies in Lao PDR have shown that the provision of high-quality molasses blocks with and without anthelmintics and 8% or 10% urea relative to large ruminants improved productivity, average daily gains, and milk production [6]. The authors argue that by including greenhouse-gas-reducing agents in molasses nutrient blocks (MNBs), an abatement of the greenhouse gas emission (GHGe) intensity of 470 kg CO_2_e per consumed MNB was obtained. Therefore, MNBs may improve global large ruminant livestock production system efficiencies.

The dietary supplementation of hydrolyzed yeast was studied as a potential source of prebiotics for alternative antibiotics in ruminants. While no impact was observed on the growth of the animals, feeding hydrolyzed yeast at a dose of 2 g/kg DM improved the digestibility of crude protein; hematological indices, especially neutrophils and monocytes; and total volatile fatty acid and propionate production in growing beef cattle [7]. The impact of hydrolyzed yeast on carcass traits and meat quality can be investigated in future studies. 

### 2.2. Monogastric Animal

The use of seeds from native trees or shrubs was investigated as a protein source for monogastric animals. Al-Harthi et al. [8] found that the oil-extracted *Moringa peregrina* seed meal (OEMPSM) is a good source of protein (27.2%) and antioxidants and has 131.4 mg/100 g of dry-weight tannic acids and 84.57% of unsaturated total fatty acids. The OEMPSM also showed considerable levels of nutrients (essential amino acids, and minerals) and has a high level of metabolizable energy (15.8 MJ/kg) that makes it a potential ingredient in poultry feeds. However, further investigations could reveal the biological feed value of the OEMPSM.

The *Moringa oleifera* Lam. meal (MOM) were used to improve the meat and bone quality of slow-growing male chickens when they have access to the outdoors [9]. The results showed that the breast muscle ash percentage was significantly greater (*p* ≤ 0.05) with 3% of MOM in the diet; however, other chemical parameters of the meat (dry matter, protein, and fat content) were not influenced by the treatments. Oleic acid (C18:1N9C) was more abundant in the breast than in the leg muscle in the treatments. Indeed, the breast muscle seems to be healthier than the leg portion with the addition of MOM to the diet. In particular, the bone ash content and phosphorous amount were improved in birds fed with 6% MOM. The results suggested that up to 6% MOM can be added to the diet with no detrimental effects on chicken meat quality while the bone quality will improve. Further studies are required on slow-growing breeds performances in outdoor conditions. 

## 3. Conclusions

In ruminants, the nutritional value of tropical grasses was assessed, and the effects of tropical herbs (*Mitragyna speciose*), plant processing (Indigo) by-products, plants extracts, and molasses nutrient blocks (MNBs) were tested with a positive impact on animal performances. In monogastric animals, Moringa seeds and meals were observed as good sources of protein in poultry feeds. Few by-products were investigated; however, tropical fruit wastes could be studied as feeds for contributing to a circular economy.
